# Monitoring of Nonsurgical Treatment of Peri-Implantitis Traditional Measurement Methods and aMMP-8 Test Technology: A Pilot Study

**DOI:** 10.1155/ijod/6681032

**Published:** 2025-11-27

**Authors:** Hanna Lähteenmäki, Ismo T. Räisänen, Taina Tervahartiala, Pirjo Pärnänen, Tommi Pätilä, Timo Sorsa

**Affiliations:** ^1^Department of Oral and Maxillofacial Diseases, University of Helsinki and Helsinki University Hospital, Helsinki 00280, Finland; ^2^Department of Pediatric Heart Surgery and Organ Transplantation, New Children's Hospital, Helsinki University, Helsinki 00100, Finland; ^3^Division of Periodontology, Department of Dental Medicine, Karolinska Institutet, Huddinge 14152, Sweden

**Keywords:** aMMP-8, nonsurgical treatment, peri-implant diseases, prevention

## Abstract

**Introduction:**

This pilot study investigated the effects of ≥6 weeks after nonsurgical treatment of the tissues surrounding dental implants, using clinical traditional methods and the aMMP-8 test technology in 39 patients.

**Methods and Materials:**

In the peri-implant pocket of 39 dental implants, an aMMP-8 concentration, gingival bleeding (BOP), and implant pocket depth (PPD), and an x-ray for radiologic bone level (RBL) were taken. The ≥6 weeks after nonsurgical treatment of the implants was carried out, and the treatment response was evaluated again after 30 days using the same methods.

**Results:**

The ≥6 weeks after nonsurgical treatment achieved a decrease in the aMMP-8 enzyme level, BOP, and the implant pockets. After 1 month, no statistically significant positive changes were observed in the result of the BOP (*p*=0.02) or the depth of deepened implant pockets (*p*=0.02) without the effect of surgical treatment, but there was a statistically significant reduction in the levels of the aMMP-8 chair-side test (*p*=0.001).

**Conclusions:**

Signs of inflammation were found to be very common in the tissues surrounding the implant. Getting the peri-implant tissue healthy in ≥6 weeks after nonsurgical treatment with traditional nonsurgical treatment would seem to be surprisingly challenging. The applicability of the aMMP-8 enzyme test in diagnosing and reading the real-time health status of the surrounding tissues in monitoring ≥6 weeks after nonsurgical treatments provides a useful and accurate method to control the health and disease.

**Trial Registration:** ClinicalTrials.gov Identifier: NCT06408467

## 1. Introduction

Clinical measurement methods are traditionally used to examine the health status of the dental implant attachment tissue [1, 2]. Peri-implant mucositis is an inflammatory alarming condition induced by plaque in the gingival tissue, where there is no bone tissue destruction yet. By removing the biofilm at this stage, the attachment tissue will heal [3, 4]. Peri-implantitis, on the other hand, is an inflammatory condition of the tissue that has already progressed to the destruction of the surrounding bone, and its treatment requires different treatment methods depending on the situation. Based on this information, we mostly treat peri-implantitis in the same way as periodontitis. Disruption of the biofilm and removal of bacterial coverings is recommended as a conservative form of treatment. Titanium curettes, ultrasound devices, powder cleaning, laser devices, and so on, can be used as tools of submucosal surface debridement [2, 3, 5]. Low-dose doxycycline medication can also be recommended to support the treatment [6]. The use of chlorhexidine is still preferred, although there is no strong evidence that it improves the treatment outcome [7, 8]. However, it is often necessary to perform surgical treatment, either resective or regenerative surgery [4, 9]. To assess the rate of progression of peri-implant diseases, the chair-side test ImplantSafe has been developed, which is based on aMMP-8, a biomarker of incipient inflammation [10, 11]. In this pilot study, the effect of ≥6 weeks after nonsurgical treatment of peri-implant health was evaluated using traditional measurement methods and the aMMP-8 peri-implant sulcular fluid point-of-care test/chairside test.

To stop peri-implant mucositis or peri-implantitis, it is necessary to diagnose them. Diagnosis is made by gingival pocket measurement, which requires precision due to the shape of the different implants [4, 9]. Bleeding on probing is a usual sign of an active inflammatory reaction that has started, but due to the deep of the gingival pocket and the sensitive measuring technique, invasion occurs [12].

There are many classifications of the depth of the gingival pocket to diagnose peri-implant mucositis or peri-implantitis. Different limit values can be used in the classifications, including, for example, Finland's treatment recommendation, where peri-implantitis is defined as BOP and PPD ≥ 6 mm and based on radiological findings, alveolar bone loss (radiologic bone level [RBL]) ≥ 3 mm [13]. Natto et al. [14] consider the limit value of peri-implantitis to be a gingival pocket ≥ 3 mm and bone loss ≥ 2 mm, while Hentenaar et al. [15] consider BOP positivity and suppuration, bone loss ≥ 2 mm as peri-implantitis compared to healthy. In Renvert et al.'s [16] classification, the definition of peri-implantitis is BOP positivity, suppuration and PPD ≥ 6 mm and bone loss ≥ 3 mm from the estimated implant insertion depth. In the classification, it is pointed out that the visual signs of inflammation can vary, and the shape of the mucosa at the implant site must be considered when evaluating the measurement depth. In addition to clinical examinations, visual signs and X-ray imaging, a chair-side test has been developed. It gives a numerical value of the biomarker in the gingival pocket and implant pocket fluid.

**Figure 1:**
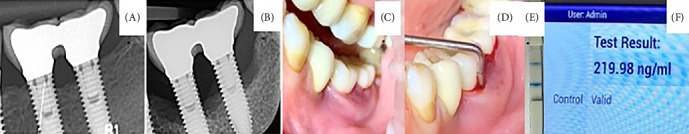
Reg. DD 36,37 Nobel Biocare implants. (A) Implants operated in 2007, and peri-implant mucositis started in 2018. (B) 2021 Formation of peri-implantitis. (C) Clinical image 2021. (D) Clinical image of gingival pocket measurement with PPD > 6 mm. (E) ImplantSafe test reading window (two strong test line). (F) ImplantSafe test reading window from the ORALyzer reader where the numerical value exceeds > 20 ng/mL.

A biomarker is a factor or characteristic that manifests a change in the biological state of an organism, and they are used to show, for example, the exposure of the organism or the environment to a foreign substance [17]. The aMMP-8 enzyme is a detectable and measurable proteolytic and collagenolytic enzyme. The presence of aMMP-8 enzyme means that the host response and defense state of the gingival tissue have been activated. Elevated aMMP-8 manifests the progressive proteolytic inflammatory state of the tissues as well as competent collagenolytic activity and can be assumed to indicate a risk for the development and progression of peri-implantitis [18]. The test is technically the same quick test as the classic pregnancy or COVID-19 antigen tests. As a diagnostic tool, it can be used to show the health status of the gingival tissue both visually by looking at the reading window of the test stick or by using the test tray in the ORALyzer reader, which gives a numerical result in ~5 min (Figure 1). The threshold values for classification between health and disease is 20 ng/mL. In peri-implant diseases, the level of aMMP-8 increases as the inflammatory condition progresses. In healthy implant tissues, the level is <20, while in early-stage and inflamed tissues, it is ≥20.

The hypothesis of this study was no-surgical professional cleaning can reduce the signs of inflammation, and the aMMP-8 test can be used to measure and monitor the health status of peri-implant tissues and monitor the possible success or failure of peri-implant mucositis and peri-implantitis ≥6 weeks after nonsurgical treatment.

## 2. Materials and Methods

For the study, 39 people were randomly selected from among the patients coming for dental hygienists ≥6 weeks after nonsurgical treatments. The study was conducted in 2022. Maintenance visits were recommended for these implant patients at 6-month intervals according to the clinic's protocol. The patients signed a study permit before coming to the treatment room. The inclusion criteria were (1) implant patients arriving for a maintenance visit at a dental clinic (Hammasklinikka Kruunu, Tampere, Finland); (2) a diagnosis of peri-implant healthy, peri-implant mucositis, or peri-implantitis by a dentist according to the definitions described below; and (3) written informed consent. The exclusion criteria included (1) smoking; (2) age under 25; (3) antibiotic treatment within 6 months; (4) the presence of a major physical limitation or restriction that prohibits the hygiene procedures used in the study protocol. A single implant with a screw crown in each patient was selected for the study. This study has complied with STROBE guidelines protocol. This clinical study was conducted in accordance with the guidelines of the Declaration of Helsinki and approved by the Ethics Committee of Helsinki University Hospital (106§/26.06.2019; dnro HUS/1271/2019). Informed consent was obtained from all subjects participating in the study.

All implants were Nobel Biocare brand screw-fixed implants. At the beginning of the treatment, the study subjects underwent the ImplantSafe test randomly from any implant, and the test was taken from the peri-implant sulcus fluid (PISF). The aMMP-8 test was performed by gently rinsing the tissue around the implant teeth with water, after which it was lightly dried. According to the manufacturer's instruction, a sterile paper test strip was placed into the buccal pocket. The test strip was allowed to remain in place for 30 s, after which it was placed in an elution tube for 5 min. The dipstick was immersed into the elution fluid for 15 s. The test strip was then inserted into the OraLyzer device, which provides a quantitative result in approximately 5 min. The year of installation of the implant varied between 1997 and 2017.

After the aMMP-8 test, PPD was measured at six measurement points, and if the measurement showed a PPD > 3 mm at one of these points, the test was marked as PPD positive (+). The BOP was registered by measuring around the implant from six different measurement points, and if there was bleeding at any point, the BOP was measured as positive (+). Periapical imaging was used to assess alveolar bone loss. The limit of peri-implantitis bone loss was set to ≥2 mm from the bone level at the time of the initial implant placement. Based on clinical health status and radiography, patients were diagnosed as having no radiographic findings, no BOP, or PPD > 3 mm in healthy implants. Peri-implantitis mucositis had no radiographic findings, but BOP positivity or PPD < 3 mm or both. In peri-implantitis, X-ray findings were ≥2 mm from the estimated bone line, either BOP positivity or PDD > 3 mm or both, and possible suppuration. Clinical photographs were taken. After the measurements, the patients were given nonsurgical treatment by a dental hygienist. The results of the treatment were checked after 30 days, first performing the ImplantSafe test and measuring bleeding and peri-implant pockets. Basic information collected from the patients is shown in Table 1.

### 2.1. Nonsurgical Treatment

The examination of the attachment tissue (BOP and PPD) was performed lightly with a periodontal probe WHO, AEEP23/WHOBX (American Eagle manufacturing Co., USA). Nonsurgical treatment refers to basic treatment, which consists of a health discussion, engaging the patient as part of the treatment team and the implementation of the treatment. In addition, we provide professional guidance of the cleaning technique for teeth and implants, as well as the removal of biofilm factors. An EMS-Piezo ultrasound device was used for the entire dentition, and a suitable plasticized implant tip was used for the dental implants. Curettes intended for natural teeth (American Eagle manufacturing Co., USA) were used for dental cleaning. An EMS Air Flow powder blower was used to clean the dentition and implants, using erythritol-based cleaning powder, and a Perio-Nozzle disposable tip was used in the deepened gum pockets of the implants. A motivational discussion about self-care was held with the patients. The patients were instructed in careful self-care using an electric toothbrush and interdental brushes. Patients were not given CHX mouthwash or antibiotics to support self-care.

### 2.2. Statistical Analyses

Analysis of variance one-way ANOVA was used for patient age and the Kruskal–Wallis test for testing differences between implant diagnosis groups. Differences in categorical and continuous variables between the healthy/peri-implant mucositis/peri-implantitis groups were analyzed using Fisher's exact and Kruskal–Wallis tests. Differences between measurements of BOP, PPD, and X-ray findings and aMMP-8 were examined by repeated-measures analysis of variance. A two-tailed *p*-value of less than 0.05 was considered statistically significant. All statistical calculations were performed with SPSS Statistics, version 27 (IBM Corp., Armonk, NY, USA) for Macintosh.

## 3. Results and Discussion

### 3.1. Start Point

Of the implants, 20 were in the upper jaw and 19 in the lower jaw.

The average of the aMMP-8 enzyme test was 50.06 ng/mL in all of them at the beginning.

In the initial situation before the start of treatment, according to traditional measurement methods, 14 (36%) patients were diagnosed with healthy implant-adjacent tissues, 16 (41%) patients with peri-implant mucositis, and 9 (23%) patients with peri-implantitis.

Overall, BOP positivity was measured (*n* = 24) in 61.5% of the implants. Deepened gingival pockets ≥3 mm were measured (*n* = 14) in 35.9% of the adjacent tissues of the implants, and X-ray findings with alveolar bone loss ≥ 2 mm from the estimated bone edge (*n* = 9) in 23%. In total, 14 patients (35.90%) had a negative test result (-) <20 ng/mL measured with the aMMP-8 enzyme test, and 25 patients had a positive result (64.10%).

### 3.2. After Nonsurgical Treatment

aMMP-8—the average of the enzyme test in all final conditions was 35.9 ng/mL. 30 days after treatment, healthy adjacent tissues of the implants were diagnosed in 17 patients (43.7%), peri-implant mucositis in 13 (33.3%) patients, and peri-implantitis in 9 (23%) patients. After nonsurgical treatment, healthy subjects (*n* = 17) without BOP or sunken gingival pockets also remained healthy.

In the peri-implant mucositis group, gingival bleeding was observed in (*n* = 11) 43.2%, deepened gingival pockets (*n* = 5) in 12%.

After the treatment, the aMMP-8 enzyme test was negative (-) in 17 (43.5%) and positive (+) in 22 (56.5%) patients, whereby 7% had benefited from the treatment when the results became negative. Compared to healthy implant tissues, patients with peri-implant mucositis and peri-implantitis had higher aMMP-8 levels on average, but patients with peri-implantitis had more problems achieving a positive treatment response.

**Figure 2:**
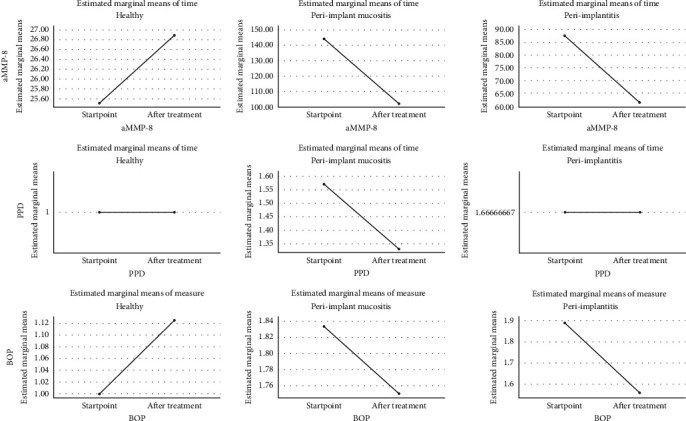
Reactions of aMMP-8 enzyme test, peri-implant pockets depth > 3 mm, and BOP (+), result in implant disease before starting treatment (start point) and after 30 days of treatment.

No statistically significant positive changes were observed in the result of the BOP (*p*=0.02) and the depth of deepened implant pockets (*p*=0.02) without the effect of surgical treatment, but there was statistically significant of aMMP-8 enzyme test (*p*=0.001) for all participations; health group (*p*=0.001), the peri-implant mucositis (*p*=0.007) and the peri-implantitis group (*p*=0.001, Figure 2).

## 4. Discussion

### 4.1. Prevention of the Treatment of Adjacent Tissues of Dental Implants

Planning the implant treatments, dentists and teams have the responsibility to evaluate both the success of the treatment from a medical point of view, as well as the patient's performance and the possibility of treating implants in the mouth. The connective tissues of dental implants differ from round natural teeth. Healing peri-implantitis without surgical treatment is not very possible, mainly due to the lost bone tissue. Therefore, preventing and stopping peri-implantitis mucositis is the most central and cost-effective goal of diagnosis and treatment. The aim of the nonsurgical treatment of periodontitis and peri-implantitis should be to achieve a periodontological healthy state in the entire mouth [19]. It should be maintenance and follow-up. When diagnosed and monitored with aMMP-8 test technology, a well-maintained treatment balance means an aMMP-8 value <20 ng/mL, that is, test negativity (−).

We found no connection or differences in how long the implants had been in the mouth or how many implants in the mouth had been operated at the same time or where the implants were in the dentition versus the test result. An elevated and positive aMMP-8 test result alarms predictively the developing peri-implantitis, which requires enhanced and therapeutic intervention. In 14 (*n* = 14) patients, the implant tissue looked healthy after clinical measurements, but in four of them, the aMMP-8 enzyme test showed an elevated value at the first measurement of 49.22 ng/mL and at the final measurement of 43.29 ng/mL. It could be that the clinical measurement technique underdiagnoses the status of the connective tissue for these implants, where the concentration of the aMMP-8 enzyme is elevated, that is, the tissue is exposed to an active collagenolytic phase slowly surety eventually leads to tissue destruction.

We also noticed that the peri-implant mucositis group had remarkably high aMMP-8 values. Clinical signs were visible in these patients, but no radiological bone destruction has yet occurred. The traditional measuring technique measures the tissue destruction that has already occurred, and the aMMP-8 test measures the current state of the tissue, so the aMMP-8 enzyme test is perfectly suited to this initial situation and its monitoring to alarm the future peri-implants diseases eventually predictively. With the help of the test, the inflammatory proteolytic and collagenolytic host response can be detected as quantitative results before the clinical signs give rise to it. aMMP-8 makes invisible predictive invisible before clinical periodontological manifestation [11, 20]. As mentioned by Renvert et al. [16], the visual signs of inflammation can vary [21], so making a diagnosis based on visual signs is also an individual perception. Reading the aMMP-8 enzyme test using a quantitative reader (ORAlyzer) gives a clear numerical value of the presence of the aMMP-8 enzyme in the PISF, leaving no room for misunderstanding. The numerical value is also understood by the patients, in which case monitoring the treatment balance is easy to understand, and it can also be helpful in motivating the patient.

### 4.2. The Effect of Nonsurgical Treatment

In our study, nonsurgical treatment mainly reduced the levels of aMMP-8 in the PISF and thus the disease severity (stage) and rate of progression and the risk profile (grade) of peri-implant diseases [11, 22]. With nonsurgical treatment in cooperation with the patient's self-care, the tissue can heal. In this study, some of the implant tissues were healthy at the beginning of the follow-up period, and this group only slightly increased from *n* = 14 to *n* = 17, that is, 7% of patients were treated from positive to negative during the 30-day treatment period with nonsurgical treatment. The aMMP-8 enzyme test result decreased on average in all patients from 89.24 to 54.5 ng/mL. BOP (from 61.5% to 41% in all patients) and PPD (from 35.9% to 28.2%) also decreased.

### 4.3. aMMP-8 Values During ≥6 Weeks After Nonsurgical Treatment Follow-up

Previous studies have shown that the negativity of aMMP-8 value is easier to achieve in the treatment of periodontitis [18], showing that the treatment and maintenance of peri-implantitis is more challenging than the treatment of periodontitis [23, 24]. The results of our study also point to this. It is noteworthy that in healthy subjects aMMP-8 and BOP measurements increased after treatment. This finding suggests that perhaps in healthy, the measurement methods and cleaning momentarily raise the host's response.

In our study, the follow-up and nonsurgical maintenance study of peri-mucositis/-implantitis, the treatment mainly reduced elevated (+; ≥20 ng/L) aMMP-8 levels in the implant pocket fluid, but not always to negative levels in healthy/treatment balance (-; <20 ng/mL). These aMMP-8 test-positive patients were identified as patients at increased risk of peri-implant disease. An elevated aMMP-8 value is ≥20 ng/mL. aMMP-8 positivity in oral fluids reflects and predicts an increased risk for the current and future state of periodontitis and peri-implantitis, as well as the development of the diseases [23, 24]. It is worth noting that total MMP-8 (tMMP-8) measurements in oral fluids have not been able to demonstrate the presence and prediction of the diseases—but in fact quite the opposite [25, 26]. It is noteworthy that aMMP-8 is not synonymous to total MMP-8 in peri-implantitis diagnosis [26].

### 4.4. Support Treatment at Home

The treatment response of peri-implant mucositis and peri-implantitis guides the perception of the challenge of nonsurgical treatment of adjacent tissues of dental implants. This means that more than manual manipulation is needed to treat these diseases. The use of subantimicrobial or low-dose doxycycline can be used to support the treatment [26]. Low-dose doxycycline has been found to improve the clinical outcomes of periodontitis and the response to nonsurgical treatment in addition to reduce oral fluid and serum aMMP-8 [26]. Its use is not based on an antimicrobial effect but on the reduction of tissue destruction by inhibiting matrix metalloproteinases [26]. No side effects were observed with the use of this supportive medication, but according to studies, there is no evidence that it would cause antibiotic resistance [27]. No clinical studies have been conducted on the topic, and the results have been obtained in the treatment of periodontitis patients [27].

Chlorhexidine is also an aMMP-8 inhibitor [7], but its long-term use is limited. Other promising at-home treatments include new Finnish innovations like photodynamic dual-light therapy [28] or fermented lingonberry juice [29]. Promising pilot studies have been published on the aMMP-8-lowering effects of these mouthwashes in the treatment of periodontitis and peri-implantitis [28, 29]. The aMMP-8 chair-side test works well independently, but alternative combination tests, for example, with virulence factors of dysbiotic bacteria to be useful combinations to aid in diagnosis and monitoring [27]. Even more treatments are needed to manage peri-implant mucositis and especially for early diagnosis of the disease and evaluation of the progression of the disease and follow-up.

The group size in this study is small. Also, the 30-day follow-up period is a short time to measure the final improvement. Dental implants are increasing in the population all the time, and it would be interesting for a researcher to see if dental implants in different parts of the mouth have different measurement results. More studies are needed to bring more information and confirm the results.

## 5. Conclusions

Keeping the peri-implant tissues healthy requires careful self-care and regular maintenance. ≥6 weeks after nonsurgical treatment by professionals can be monitored using traditional clinical methods but also with aMMP-8 test technology.

## Figures and Tables

**Table 1:** 1 Fisher's exact test was used to test gender, diabetes, asthma, rheumatism, and heart disease.

Classification	Healthy (*n* = 14)	Peri-implantmucositis (*n* = 16)	Peri-implantitis (*n* = 9)	*p*-Value
Genre	—	—	—	0.25
Man	6	4	1	—
Woman	8	12	8	—
Age mean ± SD	74.5 ± 9.4	73.2 ± 9.9	68.6 ± 6.3	0.31
Min–max	60–92	51–88	57–77	—
Implant insertion	—	—	—	0.92
Mean ± SD	2009 ± 6.7	2009 ± 6.7	2011 ± 3.6	—
Min–max	1997–2017	1997–2009	2004–2015	—
Diabetes	—	—	—	0.31
Yes	2	0	1	—
No	12	16	8	—
Asthma	—	—	—	0.42
Yes	1	3	0	—
No	13	13	9	—
Rheumatic	—	—	—	1.00
Yes	1	2	1	—
No	13	14	8	—
Heart diseases	—	—	—	1.00
Yes	5	6	4	—
No	9	10	5	—

*Note:* Analysis of variance one-way ANOVA was used to test group comparisons of patient age and Kruskal–Wallis test of implant age. Mean: average.

Abbreviation: SD, standard deviation.

## Data Availability

The data that support the findings of this study are available from the corresponding author upon reasonable request.
